# Rapid turnover of effectors in grass powdery mildew (*Blumeria graminis*)

**DOI:** 10.1186/s12862-017-1064-2

**Published:** 2017-10-31

**Authors:** Fabrizio Menardo, Coraline R. Praz, Thomas Wicker, Beat Keller

**Affiliations:** Department of Plant and Microbial Biology, University of Zürich, Zollikerstrasse 107, 8008 Zürich, Switzerland

**Keywords:** *Blumeria graminis*, Powdery mildew, Effectors

## Abstract

**Background:**

Grass powdery mildew (*Blumeria graminis, Ascomycota*) is a major pathogen of cereal crops and has become a model organism for obligate biotrophic fungal pathogens of plants. The sequenced genomes of two *formae speciales* (*ff.spp.*), *B.g. hordei* and *B.g. tritici* (pathogens of barley and wheat), were found to be enriched in candidate effector genes (CEGs). Similar to other filamentous pathogens, CEGs in *B. graminis* are under positive selection. Additionally, effectors are more likely to have presence-absence polymorphisms than other genes among different strains.

**Results:**

Here we identified effectors in the genomes of three additional host-specific lineages of *B. graminis* (*B.g. poae, B.g. avenae* and *B.g.* infecting *Lolium*) which diverged between 24 and 5 million years ago (Mya). We found that most CEGs in *B. graminis* are clustered in families and that most families are present in both reference genomes (*B.g. hordei* and *B.g. tritici*) and in the genomes of all three newly annotated lineages. We identified conserved protein domains including a novel lipid binding domain. The phylogenetic analysis showed that frequent gene duplications and losses shaped the diversity of the effector repertoires of the different lineages through their evolutionary history. We observed several lineage-specific expansions where large clades of CEGs originated in only one lineage from a single gene through repeated gene duplications. When we applied a birth-death model we found that the turnover rate (the rate at which genes are deleted and duplicated) of CEG families is much higher than for non-CEG families. The analysis of genomic context revealed that the immediate surroundings of CEGs are enriched in transposable elements (TE) which could play a role in the duplication and deletion of CEGs.

**Conclusions:**

The CEG repertoires of related pathogens diverged dramatically in short evolutionary times because of rapid turnover and of positive selection fixing non-synonymous mutations. While signatures of positive selection on effector sequences are the expected outcome of the evolutionary “arms race” between pathogen and plant immune system, it is more difficult to infer the mechanisms and evolutionary forces that maintained an extreme turnover rate in CEG families of *B. graminis* for several millions of years.

**Electronic supplementary material:**

The online version of this article (10.1186/s12862-017-1064-2) contains supplementary material, which is available to authorized users.

## Background

**Fig. 1:**
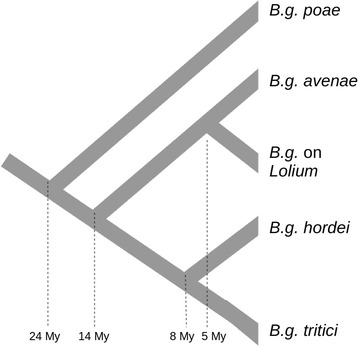
Phylogenetic tree of lineages of *B. graminis*. Simplified phylogenetic tree of the five lineages of *B. graminis* used in this study (modified from Menardo et al. [22]). The median estimation for the divergence time is reported at each bifurcation of the tree. *B. graminis* growing on *Lolium* has a different notation because it was never formally designated as a *f.sp*.

Pathogens are one of the most prominent causes of yield loss in agriculture. Fungal, bacterial, oomycete and animal infestations are generally controlled with chemical treatments and with breeding of resistant varieties. Effectors are secreted proteins produced by most pathogens and play a fundamental role in host infection. They contribute to the suppression of pathogen-triggered immunity and modulate the cellular environment, making it suitable for continued infection and nutrient uptake [1–3]. Effectors can be either apoplastic or cytoplasmic according to their localization. Both types can be recognized by plant receptors and cause effector-triggered immunity, which includes defense responses like hypersensitive cell-death response (HR)] [1, 4]. In agriculture this resistance is often rapidly overcome by new strains that, according to the classical gene-for-gene model, either lost the recognized effector(s) or carry modified allele(s) that are not recognized [5, 6]. While the genomes of most pathogenic bacteria code for only a few dozens of effectors, hundreds of putative effectors have been identified in the genomes of pathogenic oomycetes, fungi, as well as aphids and nematodes [7–11]. In fungi and oomycetes, this effector proliferation has been observed mostly in large genomes that are rich in transposable elements [8, 12–14]. It is often assumed that flexible genomes and large redundant effector repertoires have a great adaptive potential which provided an evolutionary advantage in the genetic arms race with the plant immune system [13]. Moreover, it has been shown that the genomes of some fungal and oomycete pathogens are composed of two types of compartments: gene-rich regions with “slow” evolution, and highly repetitive regions with fewer genes which show more mutations and structural polymorphisms in comparative analyses (the two-speed genome concept) [13, 15]. Although not every pathogen genome has the typical architecture postulated by the two -speed genome concept, signatures of fast evolution have been observed in effector genes of most pathogens. In particular positive selection, recombination, gene duplications and losses have been shown to be the determinants of the fast evolution of effectors in different filamentous pathogens [10, 14, 16–21]. However, these patterns have mostly been analyzed in comparative studies between closely related species which diverged less than 1 Mya or among different strains of the same species. Here we use the grass powdery mildew (*Blumeria graminis, Ascomycota*), a major pathogen of cereals and grasses, to study the evolution of effectors in five lineages infecting different grasses and cereals (wheat, barley, oat, *Lolium* and *Poa*) through more than 20 million years of evolution. In a previous study [22] we reconstructed the evolutionary history of these five lineages based on whole-genome data and found that the first lineage to diverge was the *forma specialis* (*f.sp.*) *poae* (infecting *Poa*) about 24 Mya. The diversification of the other lineages occurred later with the most recent divergence about 5 Mya between *B.g. avenae* (infecting oat) and *B. graminis* infecting *Lolium* (Fig. 1). This reconstruction is questioned by the so-called holocene hypothesis. According to this alternative hypothesis, forms of *B. graminis* that infect crops diverged very recently, with the onset of agriculture, about 10,000 years ago [14, 22–27]. In this study we adopted the divergence time estimation of Menardo et al. [22]. The genomes of two of the five lineages used in this study (*B.g. hordei* infecting barley and *B.g. tritici* infecting wheat) were found to contain hundreds of CEGs that account for about 7% of all coding genes [10, 12, 14, 28]. CEGs in *B. graminis* cluster in large families and have been found to be under positive selection [10, 14]. Moreover, comparing different strains of *B.g tritici* [14] and *B.g. hordei* [26], it was found that CEGs are more likely to be deleted in one of the isolates compared to other genes. Here we identified CEGs with a bioinformatic pipeline in the genomes of all five lineages of *B. graminis* (*B.g. hordei*, *B.g. tritici*, *B.g. poae*, *B.g. avenae* and *B.g.* infecting *Lolium*) and found that most CEG families are present in all of them with multiple members. In addition, the phylogenetic analysis showed that gene losses and duplications dramatically shaped the evolution of CEG families in the last 20 million years, altering their size and composition in the different lineages. We used a birth-death model to estimate the turnover rates in CEG families and found that it was more than 200 times higher than in other (non-CEG) gene families. These findings support the hypothesis that CEGs evolve much faster than other genes and reveal that the effector repertoire of *B. graminis* is subjected to an extremely rapid turnover.

## Methods

### Genome assemblies

The raw sequence reads of *B. graminis* lineages (*B.g. avenae*, *B.g.* on *Lolium* and *B.g. poae*) were obtained from Menardo et al. [22] (accession number SRP062198)*.* Assemblies of the latter three *ff.spp.* were performed with CLC Genomic Workbench 8 letting the software decide the word and bubble size (automatic option) Table 1.

**Table 1:** Basic statistics for the three new assembled *Bg* genomes

Lineage	Number of contigs	Assembled size	N50
*B.g. avenae*	28,511	52,497,740	5645
*B.g. on Lolium*	51,877	67,455,197	4840
*B.g. poa*	74,353	86,469,665	3777

### Gene annotation in *B. graminis*

**Fig. 2:**
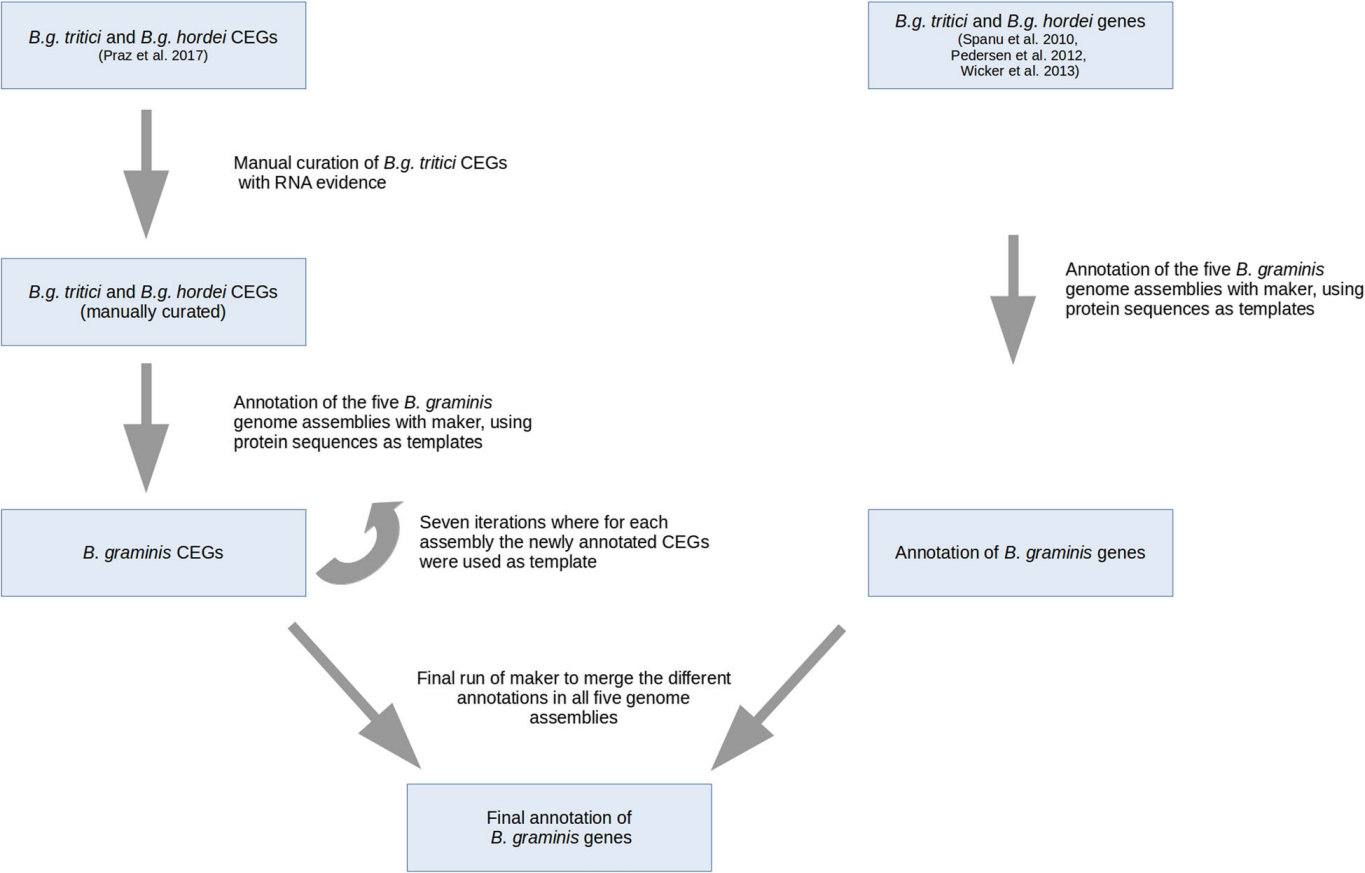
Bioinformatic pipeline for the annotation of *B. graminis* genome assemblies

We used the 1189 effectors (597 in the *B.g. tritici* reference genome (accession number PRJNA183607) and 592 in the *B.g. hordei* reference genome (accession number GCA_000151065.1) identified by Praz et al. [28] to annotate effectors from *B. graminis* infecting *Avena sativa (f.sp. avenae)*, *Poa* and *Lolium.* To improve the quality of the annotation we curated the gene models of the 597 *B.g. tritici* effectors, comparing the gene models with RNA-seq data [14], on the genome browser IGV [29]. This resulted in the reannotation of 352 genes. We used the annotation pipeline Maker 2.31.8 [30], to annotate effectors in the assemblies of *B.g poae*, *B.g. avenae* and *B. graminis* growing on *Lolium* (Additional file 1). The reference genomes of *B.g. tritici* and *B.g. hordei* had been annotated with different pipelines [12, 14]. Therefore, to avoid possible artifacts in gene content analysis we re-annotated the reference genomes with the same pipeline used for the other lineages. We ran seven iterations of Maker, the first used the *B.g. hordei* and *B.g. tritici* effectors (after manual re-annotation) identified in Praz et al. [28] as templates for the annotation of effectors in all assemblies. We used the *B. graminis* repeat database [14] (integrated in TREP, botinst.uzh.ch/en/research/genetics/thomasWicker/trep-db.html) to mask repeats during the annotation. The newly identified effectors of all *ff.spp.* were pooled and added to the templates for the next iteration. After seven iterations, we reached saturation and did not annotate any further gene. We then excluded all genes shorter than 200 bp. We (re-) annotated non-effector genes in *B.g. tritici*, *B.g. hordei*, *B.g. poae*, *avenae* and in *B. graminis* infecting *Lolium* with Maker 2.31.8 [30] with the same pipeline used for effectors but without iterations: genes were annotated using the protein databases of *B.g hordei* and *B.g. tritici* as templates [12–14]. The *B. graminis* repeat database was used to mask the genome assemblies. Finally we ran Maker 2.31.8 one more time using all annotated proteins as templates to merge the different annotations of effectors and other genes. The workflow is shown in (Fig. 2).

### CEGMA analysis

To compare the completeness of the annotations of the different genome assemblies the 458 core eukaryotic genes of *Saccharomyces cerevisiae* [31] were used as queries for blast searches against the protein databases of the five lineages of *B. graminis* that we annotated, and against the protein sequence databases of *N. crassa* and *P. anserina*. Genes with a blast hit with an e-value smaller than 10^−7^ were considered as present.

### Identification of orthologous gene families and effectors in *B. graminis*

CEGs in *B. graminis* are normally identified through bioinformatic pipelines, originally they were defined as secreted proteins without homology outside of the *Erysiphales* [12], however the original set of candidate effectors has been expanded including genes with homology to the genes identified in Spanu et al. [10, 12, 14]. All these pipelines identify candidate effector genes, only extensive functional studies can validate these predictions. Here we used the pipeline of Praz et al. [28] to identify CEGs and CEG families as follow: the protein databases of *Podospora anserina* [32] and *Neurospora crassa* [33] were downloaded from www.blugen.org, http://fungi.ensembl.org/Podospora_anserina_s_mat_/Info/Index and www.broadinstitute.org/annotation/genome/neurospora/MultiDownloads.html (accessed 01/03/2014). After elimination of proteins with homology to transposable element proteins in the protein division of the TREP database (PTREP, blastp e-value cutoff ≤10^–5,^ botinst.uzh.ch/en/research/genetics/thomasWicker/trep-db.html) (accessed 01/03/2014) we retained 9733 proteins for *N. crassa* and 10,604 *for P. anserina.*

Gene families are group of genes with sequence homology that evolved from a single gene through multiple duplications. To cluster proteins into families we performed all-against-all blast searches using protein sequences of *N. crassa* and *P. anserina* together with the protein sequences of all lineages of *B. graminis* obtained from Maker. Hits with e-value greater than 0.001 and alignment length shorter than 80% of the query length were excluded. Protein families were generated with the Markov cluster algorithm implemented in the software mcl 14–137 [34]. We tried different values of the inflation parameter (1.4, 2, 4 and 6). We then chose to use 1.4 for further analysis because it yielded a more conservative (low) number of predicted effectors, thus limiting false positives. We identified CEGs as members of families unique to *B. graminis* (no members in *N. crassa* or *P. anserina*) and with at least one gene with a predicted signal peptide (SignalP 4.0) [35]. We then manually inspected the list of families and their alignments and excluded four predicted effector families (55, 47, 8 and 8 genes) from subsequent analysis, because less than 15% of the genes (only one or two genes) harbored a predicted signal peptide.

### Alignments and phylogeny

All protein alignments were performed with muscle 3.8.31 [36], maximum likelihood phylogenetic trees were inferred with RAxML 8.2.9 [37] using a protein GTR model. Heterogeneity of the substitution rates among different sites was modeled with a discretized gamma distribution [38, 39]. In RAxML gaps are treated as missing data. Bootstrap support was computed with 1000 replications. The bootstrap convergence test implemented in RAxML showed that this was a sufficient number of replicates [40]. To check for the presence of known functional domains we used all effectors in a blast search against the conserved domain database CDD [41] http://www.ncbi.nlm.nih.gov/Structure/bwrpsb/bwrpsb.cgi (e-value cut-off =0.01). Phylogenetic trees were visualized with Figtree 1.4.2.

### Analysis of gene family evolution

We analyzed the evolution of gene families with CAFEv3.0 [42]. CAFEv3.0 models gene gains and losses along a phylogenetic tree with a random birth-death process. We used the following species tree (obtained from Menardo et al. [22]) with branch lengths in million years: (((*B.g. avenae*:5,*B.g. on Lolium*:5):9,(*B.g. tritici*:8,*B.g. hordei*:8):6):11,*B.g. poae*:24) (Fig. 1). Given the species tree and the sizes of gene families, CAFEv3.0 can optimize the value of the birth-death rate λ to maximize the likelihood of the model given the observed data. All optimizations were run 10 times and the value that gave the highest likelihood was chosen. We estimated the turnover rate (λ) for all CEG families (49 families) and for all non-CEG families (434 families) with at least 10 genes among all lineages and then tested whether CEG families have the same turnover rate as non-CEG families. Turnover rates of single gene families were inferred with a single optimization process each. We then used CAFEv3.0 to simulate family sizes (using the most likely value of λ found for non-effector gene families) and tested which CEG families significantly deviate from the null expectation (that the variation of the family size fits with the distribution predicted by the null model of evolution, which was estimated from the non-CEG families).

The reconciliation analysis was performed on all gene families with at least 10 genes, we then compared the results of CEG families with non-CEG families. For this analysis we used ecceTERA [43] with standard parameters, the software computes the most parsimonious number of gene duplications, losses and transfers to reconciliate the gene tree with the species tree.

### Analysis of genomic context of *B.g. tritici* genes

We used the high-quality *B.g. tritici* reference genome to compare the genomic context of CEGs and non-CEGs. For all 7825 predicted *B.g. tritici* genes, 5 kb upstream and downstream were extracted and TE annotation was performed by blastn seaches of the up- and downstream sequences against the *B. graminis* repeats contained in the TREP database. In total there are 92 known TE families in our in-house *Blumeria graminis* TE library. For this analysis we divided the 5 kb up and down stream of genes in 10 non-overlapping windows of 500 bp.

## Results

### Effector families are conserved among all *ff. spp.* of *B. graminis*

To systematically compare the effector repertoires of the different *ff.spp.* we annotated genes in the five lineages considered in this study with the Maker pipeline [30] using the protein databases of the *ff. spp. graminis* and *hordei* as templates [28]. The annotations of the different genome assemblies produced a similar number of genes for all lineages. We evaluated the completeness of the annotation with the core eukaryotic gene set of *Saccharomyces cerevisiae* and found that most of the core genes are present in the annotations of all lineages. Moreover, the completeness of the annotation is comparable with the one of other fungal genomes like *N. crassa* and *P. anserina* (Table 2)*.*

**Table 2:** Results of gene annotation and effector identification in *B. graminis*

Taxa	Annotated genes	Percentage of core eukaryotic genes annotated	Annotated effectors	Proportion of effectors in gene set
*B.g. tritici*	7073	96.1%	734	10.4%
*B.g. hordei*	6949	96.7%	722	10.4%
*B.g. on Lolium*	6322	96.5%	362	5.7%
*B.g. avenae*	6391	96.5%	408	6.3%
*B.g. poae*	6575	96.7%	572	8.7%
*N. crassa*	9733	98.6%	–	–
*P. anserina*	10,604	99.8%	–	–

To identify candidate effector genes, we used mcl [34] to cluster all the predicted proteins in families together with the protein databases of the non-pathogenic ascomycetes *Neurospora crassa* and *Podospora anserina.* The fungi *N. crassa and P. anserina* have a saprophytic life style and therefore we assume they do not have any genes related to pathogenicity. Moreover, they are phylogenetically relatively close to *Blumeria* [44]. We defined as effector families all families exclusive to *B. graminis* (no family members in *N. crassa* or *P. anserina*) and containing at least one gene coding for a protein with a predicted signal peptide (SignalP; [35]). We observed that not all CEGs were predicted to have a signal peptide (~ 38% of CEGs do not have a predicted signal peptide). This is partially due to incomplete coverage of some genes by the genome assemblies or by incomplete annotation. However, in most cases CEGs simply do not have a predicted signal peptide. It is possible that these genes are not functional, that they are not secreted and have a function within the fungal cell or that they are secreted through an alternative secretion system.

After manual curation, we identified 2798 predicted effector genes which clustered in 167 families. We found that CEG families (corresponding to ~ 83% of all effectors) contain genes from all the analyzed lineages of *B. graminis*. This finding implies that most CEG families were already present in the most recent common ancestor of all *B. graminis* lineages analyzed in this study, and therefore they originated before the divergence of *B.g. poae* from the other lineages (24 Ma). This also suggests that the role of most CEGs is conserved through all lineages of *B. graminis* which infect different plants, and is not specific for a single host. Additionally, we found that the genomes of *B.g. avenae* and of *B. graminis* growing on *Lolium* contain less CEGs than the other lineages (Table 2).

### Conserved domains in effectors families

**Fig. 3:**
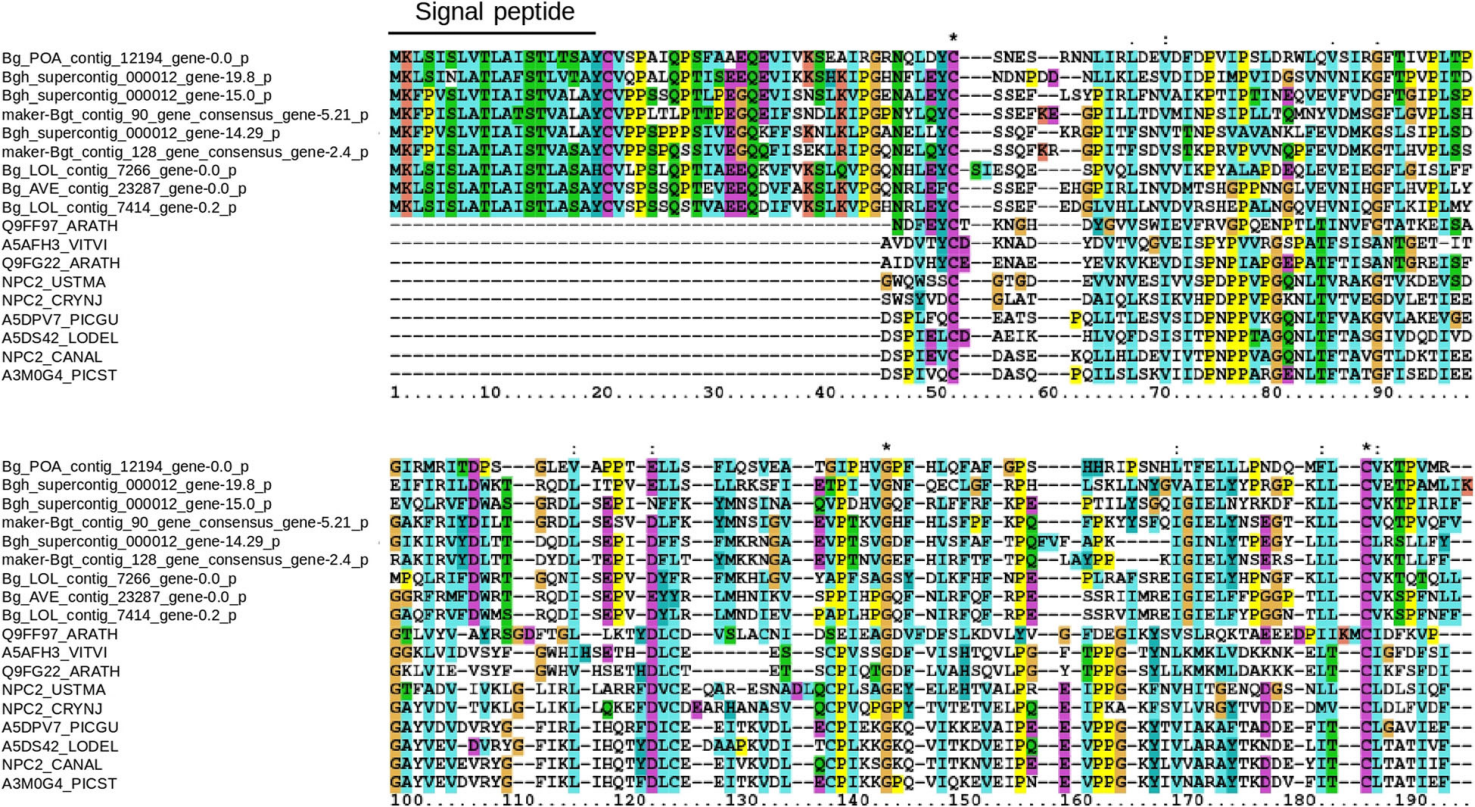
Alignment of family 17 with the ML domain. Alignment of nine representative protein sequences of family 17 with nine representative sequences of the ML domain for different species (complete alignment in Additional file 3). The alignment starts 20 amino acids after the signal peptide and covers the entire protein length of both effector proteins and ML domain sequences. Two cysteines and a glycine are perfectly conserved (indicated with asterisks). Moreover, the spacing between hydrophobic amino acids is conserved throughout the alignment (the complete alignment is available in Additional file 3). *B. graminis* genes are named according to their *f.sp.* (Bg_POA = *B.g. poae*; Bgh = *B.g. hordei*; Bgt = *B.g. tritici;* Bg_AVE = *B.g. avenae;* Bg_LOL *= B.g.* infecting *Lolium).* The members of the ML domain are named with their Uniprot ID: GENENAME_SPECIES (ARATH = *Arabidopsis tahliana*; VITVI = *Vitis vinifera*; USTMA = *Ustilago maydis*; CRYNJ = *Cryptococcus neoformans*; PICGU = *Meyerozyma guilliermondii; LODEL = Lodderomyces elongisporus; CANAL = Candida albicans; PICST = Scheffersomyces stipitis)*

We used the conserved domain database CDD [41] to identify known functional domains in all *B. graminis* CEGs. We found that only 209 CEGs code for proteins with a known domain (corresponding to the ~7.5% of all CEGs, Additional file 2: Table S1). The most represented domain was the RNAse domain (85 genes in 15 families, Pfam ID; PF00545), followed by the MD2-related lipid-recognition domain (ML) (six genes in family 17, Table 3, *Pfam ID:* PF02221). The RNAse domain was earlier described in *B.g. hordei* effectors [10, 12], while the ML domain has not yet been described in candidate effectors of *B. graminis*. However, the ML domain was found to be enriched in putative effectors of rust fungi [45]. To further analyze the homology between family 17 and the ML domain we aligned the 91 protein sequences that define the ML domain (pfam seed) with the 30 candidate effector protein sequences of family 17. Two cysteines and a glycine are conserved between all encoded proteins of family 17 and the ML domain. Furthermore, the spacing between hydrophobic amino acids is conserved through the entire protein length (Fig. 3, Additional file 3).

**Table 3:** The 20 largest families of effectors in *B. graminis*

ID^a^	Total^b^	*Bg tritici* ^c^	*Bg hordei* ^d^	*Bg avenae* ^e^	*Bg on Lolium* ^f^	*Bg poae* ^g^	SignalP^h^	Length (aa)^i^	Protein domains^j^	Fast Evolution^k^	λ^l^
1	609	161	151	113	69	115	127	288		*	80.187
2	141	30	37	22	11	41	112	153		*	14.130
3	131	9	5	12	6	99	49	317	5 microbial_RNases	*	1.901
4	103	23	50	7	8	15	74	332	16 microbial_RNases	*	0.062
5	103	29	29	14	12	19	60	323	23 microbial_RNases	*	0.048
6	101	47	40	6	8	0	95	114		*	2.298
7	91	9	21	10	11	40	61	145		*	16.931
8	68	19	14	11	20	4	38	331	12 microbial_RNases	*	0.105
9	63	16	15	8	8	16	7	253			0.027
10	62	25	12	8	8	9	44	133		*	0.095
11	52	18	17	6	7	4	45	114	2 TROVE	*	0.073
12	50	4	18	6	7	15	20	133		*	0.132
13	44	19	10	6	8	1	14	262		*	0.108
14	37	10	5	4	6	12	27	178	4 microbial_RNases		0.045
15	33	4	6	5	6	12	18	312	9 microbial_RNases		0.095
16	31	15	7	2	4	3	29	150		*	0.170
17	30	6	9	3	4	8	28	175	6 ML	*	0.029
18	27	10	5	3	4	5	25	157		*	0.037
19	26	3	10	5	6	2	18	169		*	0.069
20	26	3	4	2	3	14	11	181			0.023

^a^Family identifier

^b^Total number of genes in the family

^c^Number of family members in *B.g. tritici*

^d^Number of family members in *B.g. hordei*

^e^Number of family members in *B.g. avenae*

^f^Number of family members in *B.g.* infecting *Lolium*

^g^Number of family members in *B.g. poae*

^h^Number of genes in the family with predicted signal peptide (SignalP)

^i^Average length of the protein sequences

^j^Conserved protein domains found in the NCBI CDD (only domains found in at least 2 genes are reported)

^k^Families that evolved significantly differently (*p*-value <0.01) compared to the null model inferred overall non-effector gene families

^l^Turnover rate (average number of duplications and disruptions that a gene undergoes in a million years)

We found several additional protein domains in *B. graminis* CEGs, they are involved in a multitude of different processes and it is often difficult to relate them to pathogenicity (Additional file 2: Table S1).

### Evolution of effector families is shaped by gene duplications and losses

In a previous study [22], we reported on the evolutionary history of *B. graminis* and found that the earliest lineage to diverge was *B.g. poa* (about 24 Ma), followed about 14 Ma by a lineage that later gave rise to *B.g. avenae* and *B. graminis* infecting *Lolium* (about 5 Ma). Finally *B.g. hordei* and *B.g. tritici* diverged about 8 Ma (Fig. 1).

**Fig. 4:**
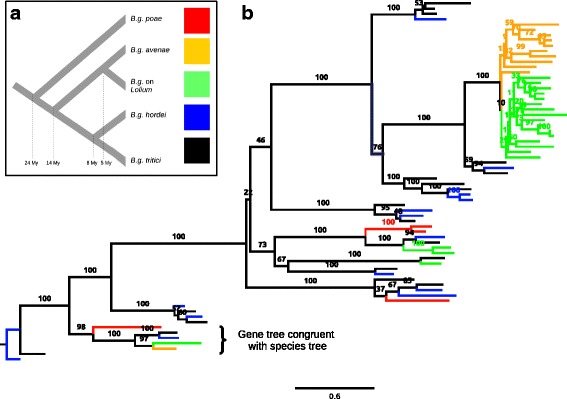
Phylogenetic tree of effector gene family 8 (**a**) Simplified phylogenetic tree of the five lineages of *B. graminis* used in this study (modified form Menardo et al. [22]). The median estimation for the divergence time is reported at each bifurcation of the tree. **b** Maximum likelihood tree of CEG family 8. Branches are colored according to the lineage of *B. graminis* to which the respective effector gene belongs. The species tree with the color code is represented in panel **a**. Branch labels report the bootstrap support for the clade inferred with 1000 replications. The scale is in expected amino-acid substitutions per site. In the lowest part of the tree there is one of the few effector genes in *B. graminis* for which we found a single copy in all lineages and for which the gene tree is concordant to the species tree. Two large lineage-specific expansions (18 genes in *B.g.* on *Lolium* and 9 genes in *B.g. poa*) are present in the upper part of the figure

**Fig. 5:**
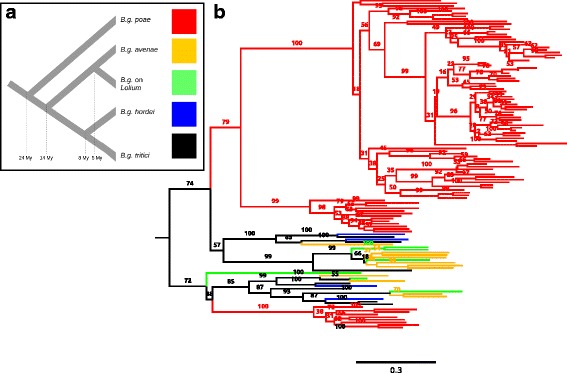
Phylogenetic tree of effector gene family 3 (**a**) Simplified phylogenetic tree of the five lineages of *B. graminis* used in this study (modified form Menardo et al. [22]). The median estimation for the divergence time is reported at each bifurcation of the tree. **b** Maximum likelihood tree of CEG family 3. Branches are colored according to the lineage of *B. graminis* to which the respective effector gene belongs. The species tree with the color code is represented in panel **a**. Branch labels report the bootstrap support for the clade inferred with 1000 replications. The scale is in expected amino-acid substitutions per site. In this family there is the most massive lineage-specific expansion: a clade composed of 90 genes found in the *B.g. poae* genome

**Fig. 6:**
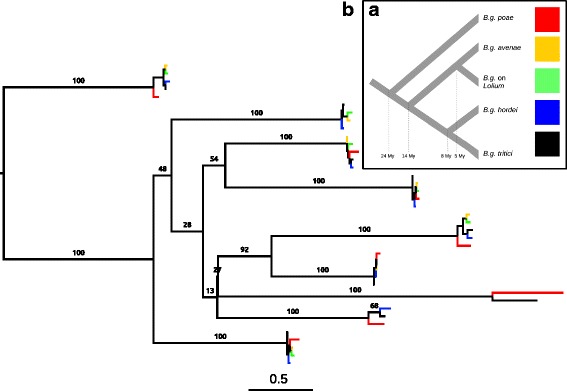
Phylogenetic tree of a transporter gene family (**a**) Simplified phylogenetic tree of the five lineages of *B. graminis* used in this study (modified form Menardo et al. [22]). The median estimation for the divergence time is reported at each bifurcation of the tree. **b**) Maximum likelihood tree of a major facilitator family (transmembrane transporters). This was randomly picked among non-effector genes families to show an example of the different patterns in the evolution of CEG families compared to non-CEG families. Branches are colored according to the lineage of *B. graminis* to which the respective effector gene belongs. The species tree with the color code is represented in panel a. Branch labels report the bootstrap support for the clade inferred with 1000 replications. The scale is in expected amino-acid substitutions per site. In this family, the average number of substitutions after divergence of the different lineages is much lower than in CEG families; this is represented by the short terminal branch lengths and could be explained by positive selection that fixes non-synonymous substitutions. Moreover, nine different genes are clearly recognizable. For five of them there is one copy for each lineage and for two of them the gene tree concords with the species tree. This is in contrast with effector gene families where it is often impossible to identify orthologous genes in the different lineages

To study how the evolution of CEGs in *B. graminis* relates to the species tree, we performed a phylogenetic analysis of all effector families using RAxML [37] to infer the maximum likelihood tree of each family. We inspected the phylogenetic trees of the 20 largest families (containing 65.5% of total effectors) and found that only 11 genes (0.6% of total CEGs; six in family 1, two in family 5, one in families 8, 16 and 19) produced sub-trees that are identical with the species tree (example in Fig. 4). In most cases it was impossible to identify a single orthologous CEG in all lineages because of multiple gene duplications and deletions that altered the gene trees (Figs. 4, 5 and 6). Gene duplications and losses also explain the large variance of family sizes among different lineages (Table 3).

**Fig. 7:**
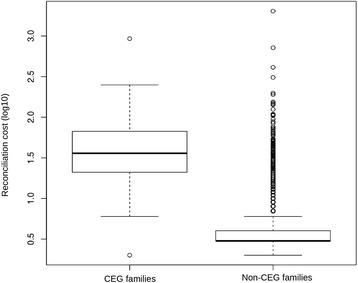
Most parsimonious reconciliation cost (log10) in CEG families and non-CEG families. Most CEG families have a higher reconciliation cost (the number of gene duplications, losses and transfers weighted by their cost) compared to other gene families

In addition, we found only 46 families for which all lineages have an equal number of genes (all of them except one have one member for each lineage). Among the 45 CEG trees with one gene for each lineage we found that only 29 (64%) are identical to the species tree. This is in contrast with the results of Menardo et al. [22] who found that, in a genome-wide survey, more than 96% of single-copy gene trees are identical to the species tree in the same five lineages of *B. graminis*. The incongruence between gene trees and species tree can be caused by incomplete lineage sorting (ILS), horizontal gene transfer, or by the presence of so-called deep paralogs (genes that originate from a common ancestor which contained multiple copies of a gene, and where different copies of the gene were deleted in different descendants). To test whether CEG gene trees are significantly less congruent with the species tree compared to non-CEGs we performed a reconciliation analysis with ecceTERA [43]. This analysis showed that a higher number of gene duplications, losses and transfers are required to reconcile CEG gene trees with the species tree compared to non-CEG gene trees (Kolmogorov-Smirnov *p*-value <2.2e-16; Fig. 7). Overall, these results indicate that gene duplications and losses had a major role in the evolution of effector families in *B. graminis.*

### Rapid turnover of effectors in *B. graminis*

**Fig. 8:**
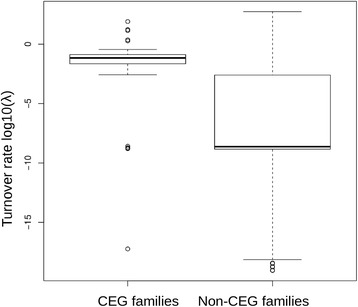
Turnover rate (log10(λ)) in CEG families and non-CEG families. Most CEG families have a higher turnover rate compared to other gene families

Variation in gene family sizes among related species can be due to random contraction and expansion caused by gene duplications and losses. Hahn and colleagues [46] developed a method that uses a birth-death model as null hypothesis (random contraction and expansion) and computes the probability that a given gene family evolved according to a different model (i.e. non-random or with different birth-death parameters). Additionally, the method of Hahn et al. [46], implemented in CAFE [42] infers the most likely value of the birth/death rate (or turnover rate λ). This rate can be interpreted as the average number of duplications and disruptions that a gene undergoes in a period of time. We found that the turnover rate over all CEG families is more than 200 times higher compared to non-CEG families (2.19 vs 0.01 per million years). However, this value can be biased by outliers, therefore we also inferred the turnover rate for every gene family singularly. Again we found that the distribution of turnover rates for CEG families is shifted towards higher values compared to non-CEG families (median effectors: 0.07 per million years, median non-effectors: 2e-9 per million years; Kolmogorov-Smirnov *p*-value <2.2e-16; Fig. 8, Table 3, Additional file 2: Table S1). Moreover, the variation of the turnover rate among different CEG families is very large, with some extreme outlier families that have turnover rates larger than ten (family 1, 2 and 7). Altogether these results indicate that CEG families overall lose and gain genes at a much faster rate than other gene families, and that this effect has a different intensity among the CEG families of *B. graminis.*

### Genomic context of effector genes in *B.g. tritici*

**Fig. 9:**
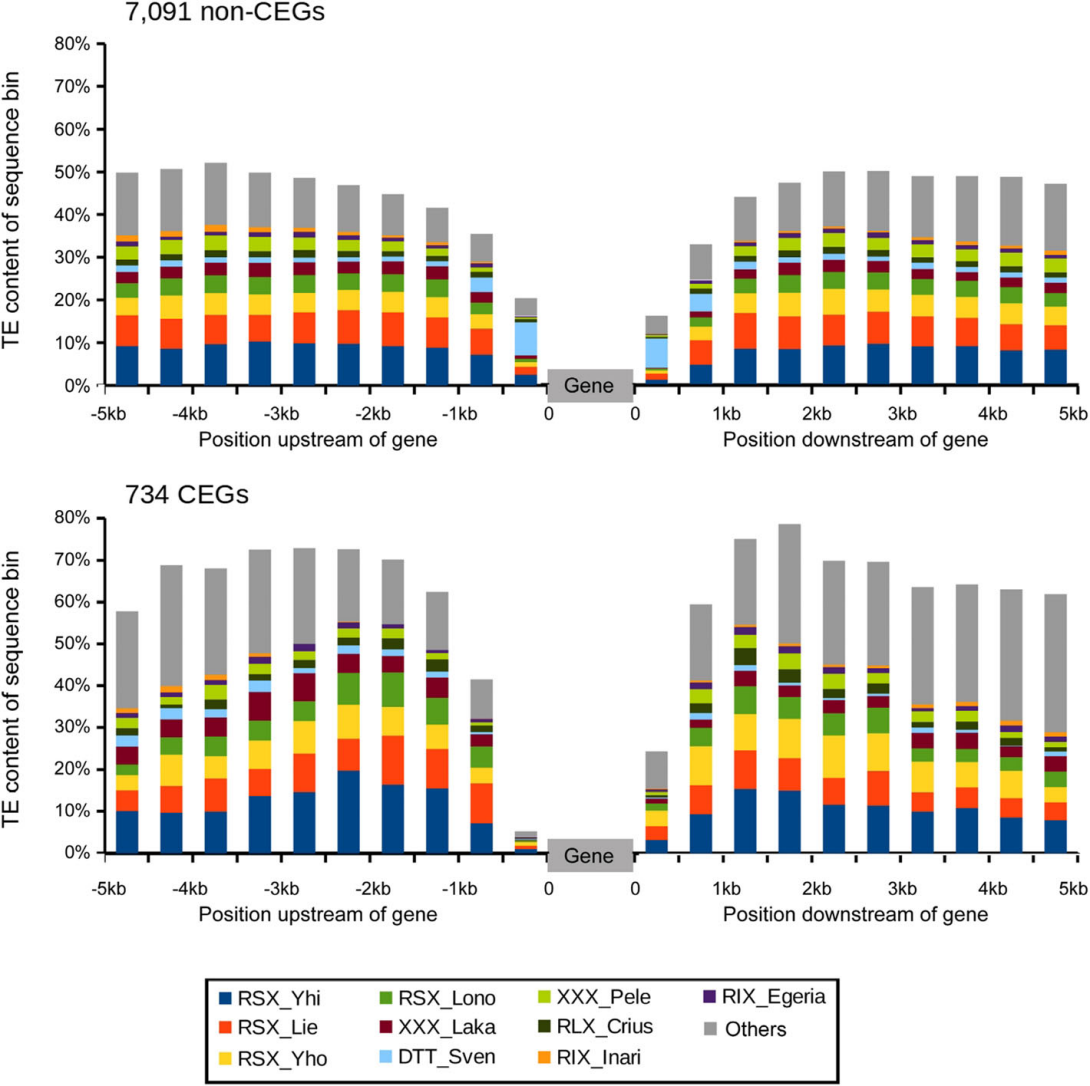
Transposable element composition of sequences surrounding *B*. *graminis* genes. Genes were separated into CEGs and non-CEGs. The 5 kb upstream and downstream of the predicted start and end point of the CDS were divided into 10 sequence bins. For each bin, average TE composition was determined across all sequences in the dataset

Rapid evolution of effector genes was associated with their occurrence in repeat-rich genomic regions in the oomycete *P. infestans* and in the fungus *L. maculans* [15]. Moreover, Pedersen et al. [10] analyzed the genomic context of a small number of predicted CEGs in the genome of *B.g. hordei* and found that some of them were surrounded by retrotransposons. Here, we focused on the high-quality *B.g. tritici* reference genome to characterize the genomic context of CEGs in comparison with non-CEGs. We used a window based approach and found that the repeat content surrounding candidate effector genes peaks at 75–80% at a distance of approximately 2 kb from the genes. In contrast, TE content at the same distance from non-candidate effectors is only 45–50% (Fig. 9). Many TE families, and especially three families of SINE, are responsible for the higher abundance of TEs in the 5 kb surrounding CEGs (Fig. 9). This could provide a possible mechanism for the duplication of these genes: if elements of the same TE family are found in the same orientation up- and downstream of a gene, they can be templates for unequal crossing overs that result in the duplication (or removal) of the sequence between the two TEs (i.e. the region containing the gene). However, unequal crossing over between TEs flanking CEG genes can also lead to removal of the genes. Previous studies reported numerous CEG presence-absence polymorphisms in different mildew isolates [14, 28]. Interestingly, we also found that the 500 bp upstream of CEGs have a remarkably low content of TE compared to non-CEGs.

## Discussion

### Orthologous effector families in *B. graminis*

In this study we predicted effector genes in silico in the genome assemblies of five lineages of *B. graminis* with divergence times between 24 and 5 Ma. This represents a unique dataset, that compared to our previous study [10, 14, 26] has an expanded taxonomical and temporal breath, and it constitutes a vintage point to study the evolutionary processes that acted on CEGs in *B. graminis* over a long period of time. We found that more than 80% of the 2798 predicted effectors belong to families that include genes from all the analyzed lineages. Moreover, it was previously found that the great majority of *B.g. hordei* and *B.g. tritici* effectors do not have homologous genes in the genomes of pea (*Erysiphe pisi*) and *Arabidopsis* powdery mildew (*Golovinomyces oronti*) [47]. These findings imply that most effector gene families of *B. graminis* originated in the period between the divergence of *B. graminis* from other mildews and the beginning of the differentiation of the different *B. graminis* lineages (22–83 Ma) [22]. The finding that effectors are grouped in orthologous families suggests that their targets are also the product of homologous genes in the different grass and cereal host species. However, we observed that effectors of different lineages belonging to the same family are often very divergent at the sequence level due to positive selection and to the lineage-specific expansions and contractions of effector families. De Guillen et al. [48] found that effector genes without sequence homology of the ascomycete pathogen *Magnaporthe oryzae* have highly similar structures. It is therefore possible that also in *B. graminis* the functionality of effectors is determined mostly by the 3D structure of the protein rather than by the amino-acid sequence.

### Conserved protein domains in effector families

Although most effectors do not have homology with known protein domains, we found that 209 of them have homology with a conserved protein domain. The most abundant was the RNAse domain which was already described in Pedersen et al. [10]. Using template-based modeling of effectors, Pedersen et al. [10] also showed that it is possible to identify a structural homology to the RNAse domain for several additional effector families in *B.g. hordei*. Three of the characterized avirulence genes (*AvrPm2* in *B.g. tritici*, *Avr*_*a13*_ and *Avr*_*a1*_ in *B.g. hordei*) and a putative suppressor of resistance (SvrPm3^a1/f1^ in *B.g. tritici*) belong to this class of RNAse-like effectors [28, 49–51]. However, Pedersen et al. [10] also found that the catalytic residues responsible for the RNAse activity are not conserved in *B.g. hordei* effectors. Additionally, we found two protein domains in *B. graminis* effectors, ML (6 genes) and CFEM (4 genes) domains, that have also been found to be enriched in the secretomes of two rust fungi (*Melampsora larici-populina* and *Puccinia graminis f.sp. tritici*) [45]. These two rust pathogens are Basidiomycetes with an obligate biotrophic life style and they both form intracellular haustoria. The finding that these domains are present in the effector sets of the distantly related pathogen species rust and mildew suggests a convergent evolution of different organisms with a similar life style. While CFEM (cystein-rich fungal domain) has not been functionally characterized, the ML (MD2-related lipid-recognition) domain is present in many animal, plant and fungal proteins and is well studied [52]. Proteins with this domain are usually shorter than 200 amino acids and involved in regulating lipid metabolism. In addition, they act as cofactors in recognition of pathogen-associated lipids and in the phospholipid transfer through membranes [52]). The well characterized protein from which the domain takes its name is MD-2. This protein interacts with Toll-like receptor 4 and it is necessary to recognize bacterial lipopeptides and lipopolysaccharides (LPS) in animals [53]. Structural homology between some plant receptor-like kinase (RLK) and animal Toll receptors suggest similarities in the modes of action [54]; moreover, the receptor-like kinase Nt-Sd-RLK was shown to respond to bacterial LPS in *Nicotiana tabacum* [55]. Among fungal membrane components, so far only ergosterol has been observed to be specifically recognized by plants [56], causing a typical elicitation response not caused by plant or animal sterols [57]. Ergosterol is a perfect PAMP candidate because it is not present in plant membranes and can be recognized as non-self by the plant immune system [58]. It is not known how plants recognize ergosterol, but one *Arabidopsis* RLK was shown to be responsive to others steroids (brassinosteroids) and to directly bind brassinolide [59]. Therefore, we propose that the ML domain could be involved in the interactions occurring between the pathogen and host membranes, either blocking the recognition of fungal lipids by the plant cell receptors or interfering with the lipid signaling system of the plant.

### Fast evolution of CEG families by gene duplication and loss

We observed that CEG families evolved following a birth-death model with an extremely high turnover rate compared to non-CEG families. This resulted in numerous gene deletions/disruptions and duplications that generated lineage-specific expansions and extinctions. To our knowledge no other study estimated the turnover rate in effector families. Pendleton et al. [60] found that in the rust fungus *Cronartium quercuum f. sp. fusiforme* (Basidiomycetes) CEG families underwent species-specific gene duplications. Moreover, Jiang et al. [61] and Goss et al. [20] reported repeated effector duplications in *Phytophtora ramorum* (Oomycetes), a pathogen with a broad host range.

The anomalous variation of CEG family sizes among lineages could be caused by natural selection or be the result of frequent structural modifications specific for CEG-containing regions. In the latter case, structural polymorphisms would be neutral and fixed by genetic drift. In *B. graminis,* effectors of the same family tend to weakly cluster in the same genomic region, but overall are found across the entire genome [10] (our own unpublished data). Based on this observation Pedersen et al. [10] proposed unequal crossing over as mechanism for repeated CEG duplications. It is possible that major structural rearrangements involved multiple members of the same family, causing fast expansions or reductions of some families (for example the CEG families 1, 2 and 7 with high turnover rate). Moreover, we found that the genomic context of CEGs has a high TE content, which could cause effectors to be duplicated and deleted more often than other genes in absence of selective pressure. The association of effectors with transposon-rich regions was observed also in other pathogens [13], and recently it was proposed that transposons have the major driving force in the adaptive evolution of the genome of fungal pathogen *Verticillum dahliae* [62].

It was shown before that CEGs in *B. gramini*s and in other pathogens are exposed to two kinds of selective forces: the first is the necessity to maintain their virulence functions through time [10, 14]. The second is caused by the plant immune system: effectors can be recognized by immune receptors, which trigger an immune response that leads to the death of the pathogen (*AvrPm3* ^*a2/f2*^ and *AvrPm2* in *B.g. tritici*, *Avr*_*a1*_ and *Avr*_*a13*_ in *B.g. hordei*) [28, 50, 51]. The effects of these two kinds of selection (escaping recognition and optimizing virulence) on the sequence of effectors are difficult to tear apart because in theory they both leave the typical signatures of positive selection [21]. Only the molecular characterization of the interactions between effectors and their targets and between effectors and resistance proteins will clarify to what extent positive selection is due to either of the two mechanisms. Conversely, gene loss is a well-documented evolutionary mechanism to escape recognition from a resistance gene in many pathogens. In powdery mildew, two different mechanisms have been reported: complete gene deletion (*AvrPm2* in *B.g. tritici*) [28] and insertion of unrelated DNA in the coding sequence (*Avr*_*a13*_) [51]. It is tempting to speculate that the fast turnover rate that we observed in effector families of *B. graminis* is the direct consequence of the selective pressure of the plant immune system. Three of the four known *Avrs* in *B. graminis* belong to families with a large size variation and a high turnover rate (the exception is *Avra1* in *B.g. hordei* which belongs to a family of only five genes that has a phylogenetic tree identical to the species tree). However, it is not clear whether selection or a neutral process (genetic drift) is the evolutionary force that drove the evolution of a high number of redundant effectors, which is a prerequisite to the possibility of losing them to escape recognition. Duplicated effectors could have given an immediate evolutionary advantage to the pathogen, impeding their molecular targets in the plant cell from evolving toward less susceptible variants: a plant target protein with a mutation that hampered the function of one effector would not have been fixed by positive selection because other slightly different effectors (resulting from a duplication and a subsequent mutation) were still functional. This is analogous to the strategy used in medicine and agriculture to prevent the evolution of drug-resistance in pathogens [63]. Alternatively, flexible genomes and specifically flexible effector repertoires could provide an increased adaptive potential to the pathogen and be themselves a trait under selection.

## Conclusion

We identified 2798 CEGs in the genome of five lineages of *B. graminis* with divergence times between 24 and 5 million years. We found that most of them cluster in families based on sequence similarity, and that most families are present in all lineages. Additionally, we found a high turnover rate in CEG families that resulted in large lineage-specific expansions and extinctions and contributed massively to the diversification of effector repertoires in lineages of *B. graminis.* Positive selection, previously described in Pedersen et al. [10] and Wicker et al. [14], and fast turnover act in parallel and are responsible for the fast evolution that we observe in effectors of *B. graminis*. These two processes caused a fast diversification of CEGs and are probably contributing to the adaptation of different lineages to different hosts. While positive selection fixes non-synonymous mutations, the fast turnover has two effects: it creates the effector repertoires on which positive selection can act (gene duplication), but at the same time destroys genes, contributing to increase the diversity of effector repertoires in pathogen populations.

## Additional files

Additional file 1: 1) Genome assembly of B.g. poae (Bg_POA). 2) Genome assembly of B.g. avenae (Bg_AVE). 3) Genome assembly of B.g. growing on Lolium (Bg_LOL). 4) CDS databases of the 5 ff.spp. analyzed in this study (5 files: *all_CDS_last_annotation_200). 5) file with all effector families in B.g. Each line correspond to a family and all member are listed. The gene names correspond to the name in the CDS databases (candidate_effector_gene_families). (ZIP 72153 kb)

Additional file 2: Table S1. Information on effector families in *B. graminis*. (XLSX 27 kb)

Additional file 3: Protein alignment of familiy17 with the ML domain seed (FASTA 4 kb)
